# Biological Monitoring of Hexavalent Chromium and Serum Levels of the Senescence Biomarker Apolipoprotein J/Clusterin in Welders

**DOI:** 10.1155/2008/420578

**Published:** 2008-04-22

**Authors:** Evangelos C. Alexopoulos, Xenophon Cominos, Ioannis P. Trougakos, Magda Lourda, Efstathios S. Gonos, Vassilios Makropoulos

**Affiliations:** ^1^Department of Public Health, Medical School, University of Patras, 26500 Rio Patras, Greece; ^2^Hellenic Institute for Occupational Health and Safety, 6 Thirsiou Street, 10445 Athens, Greece; ^3^National Hellenic Research Foundation, Institute of Biological Research and Biotechnology, 48 Vas. Constantinou Avenue, 11635 Athens, Greece

## Abstract

Welding fumes contain metals and other toxic substances known or strongly suspected to be related with oxidative stress and premature cellular senescence. Apolipoprotein J/Clusterin (ApoJ/CLU) is a glycoprotein that is differentially regulated in various physiological and disease states including ageing and age-related diseases. In vitro data showed that exposure of human diploid fibroblasts to hexavalent chromium (Cr(VI)) resulted in premature senescence and significant upregulation of the ApoJ/CLU protein. In this study we analyzed blood and urine samples from shipyard industry welders being exposed to different levels of Cr(VI) over a period of five months in order to assay in vivo the relation of ApoJ/CLU serum levels with Cr(VI). Our findings confirmed the previously reported in vitro data since reduction of Cr levels, after a worksite intervention, associated with lower levels of ApoJ/CLU serum levels. We concluded that the human ApoJ/CLU gene is responsive to the acute in vivo oxidative stress induced by heavy metals such as hexavalent chromium.

## 1. INTRODUCTION

Welders are exposed to many air contaminants such as iron oxide, manganese, nickel, cadmium oxide, zinc oxide, chromium, fluoride, ozone, nitrogen oxides, carbon monoxide, and others [1]. Previous studies suggested that the increased generation of highly reactive oxygen species, which result in oxidative tissue damage, is responsible for the toxicity of Cr(VI), Fe, Ni, and other metals [2–6]. Welding processes, like manual metal finishing, are commonly used in stainless steel welding and produce mainly chromium, nickel, manganese, fluorides, nitrogen oxide, and ozone [1].

The genotoxic, mutagenic, and cytotoxic effects of hexavalent chromium (Cr(VI)) exposure are well documented [2–6]. Chromium is absorbed via the gastrointestinal and respiratory tracts and the skin. Even though chromium kinetics is not fully clear, key features mainly include differential absorption of Cr(VI) and Cr(III), rapid reduction of Cr(VI) to Cr(III) in all body fluids and tissues, modest incorporation of chromium into bone, and concentration- dependent urinary clearance [7]. Compared to the Cr(III) ions that cross the membranes slowly by simple diffusion, Cr(VI) readily crosses cell membranes in the form of tetrahedral chromate anions through the general anion transport system and for that reason intracellular Cr is considered as indicative of Cr(VI) exposure [8–10]. Inside the cell, Cr(VI) is reduced to Cr(III), generating intermediate Cr(V) and Cr(IV) ions, oxygen, and organic radicals. The existing evidence points to Cr(V) as the main reactive species in Cr(VI)-induced genotoxicity [11, 12] through direct redox reactions with DNA, formation of DNA adducts, and Zn(II) thiolate complexes [13] which, in addition to Cr(VI) complexes formed mainly with cellular thiols, are likely triggers of a chain of events leading to carcinogenesis [14]. Products including DNA strand breaks, Cr-DNA adducts, and DNA-protein cross-links have been shown to occur in vivo and in vitro [15, 16].

The distribution of chromium to different compartments, the possibility of different transport mechanisms and pathways combined with the potential for reduction of Cr(VI), complicates further the kinetic models as far as it concerns excretion [17, 18]. The decrease of Cr(VI) levels from body fluids seem to follow a biphasic blood clearance and a bi/multiphasic urinary excretion pattern, which suggest the existence of several slow-releasing storage compartments. Several studies have shown half-times ranging between a few days (2–6) up to more than 3 months or even two years for the fast/medium and slow phase elimination, respectively [19–21].

Recently reported data suggested that exposure of human diploid fibroblasts to hexavalent chromium Cr(VI) at concentrations equal or 10 fold lower than the maximum permissive values (MPV) resulted in cell death or premature cellular senescence, respectively [22]. The cellular senescence phenotype was accompanied by elevated protein levels of apolipoprotein J/clusterin (ApoJ/CLU) [22].

Human ApoJ/CLU is a heterodimeric secreted glycoprotein that was initially purified from serum and identified as an apolipoprotein [23]. Not only ApoJ/CLU functions as an apolipoprotein, but it is also implicated in additional intra-or extracellular processes. For instance, it has been proposed that the secreted ApoJ/CLU protein functions as an extracellular chaperone [24]. CLU is differentially regulated in many severe physiological disturbance states including ageing, several neurological diseases, and in vivo cancer progression [23].

Interestingly and surprisingly, in a cross-sectional field survey, it has been also found that welders and sandblasters (known to be exposed to high levels of heavy metals and other chemicals) exhibited lower ApoJ/CLU serum levels as compared to other low chemically exposed occupational groups like white collars and electricians [22]. Given these observations in this study we analyzed blood and urine samples from shipyard industry welders being exposed to different levels of Cr(VI) over a period of five months in order to assay in vivo the relation of ApoJ/CLU serum levels with Cr(VI).

## 2. MATERIALS AND METHODS

### 2.1. Sample collection

Blood and urine samples were collected from male welders (*n* = 75) and sandblasters (*n* = 5) of a shipyard industry according to standard procedures. Subjects aged between 22 to 58 years old (mean 40.14) had worked for 2 to 35 years in the shipyard (mean 18.5) and agreed to participate in this study after signing an informed consent. The male welders examined in this study were selected among welders who welded on both MS and SS. Welding has taken place inside workshops or SS tanks. Welders used electrodes containing various metals in different concentrations like Mn (0.8–6.5%), Ni (0.02–8.8%), Cr (0.03–22.5%), Mo, Si, Fe, Zn, Cu, and other substances. All welders had access to local suction at their workplace and used it more than 75% of the welding time. None wore airstream helmet, but all occasionally wore filter mask for personal respiratory protection and consistently used protective clothing and gloves. Each participant completed a comprehensive questionnaire on individual welding history and on welding methods and intensity applied during the previous two weeks, month, and the previous year. They were also asked about their welding employment histories and the duration of SS welding in their careers. As determined after detailed medical examination, none of the subjects suffered any serious chronic disease.

All welders with Cr blood levels above 2 *μ*g/L (*n* = 9) were selected to enter an intervention phase. This worksite intervention aimed to lower Cr(VI) exposure through a minimization of stainless steel welding. Five months later, sample collection was repeated in this selected group of workers. The five months period was selected based on half time of chromium life and life cycle of erythrocytes.

In parallel to blood and urine collection, a number of additional parameters were recorded such as age, anthropometrical characteristics, smoking status, duration of employment, medical history, and detailed occupational history.

**Measurement of Cr and ApoJ/CLU levels in blood and urine samples**

Cr levels in blood and urine samples were measured by using a Perkin-Elmer 600 atomic absorption spectrometer. Samples were appropriately diluted (1/2 for urine and 1/5 for blood) with Triton X-100 in a nitric acid solution. They were then analyzed by the standard addition calibration procedure using graphite furnace [25] at a detection limit of 0.1 *μ*g/L. to correct the differences in fluid intake, the urinary values were also related to the respective creatinine values. Quantitative measurement of ApoJ/CLU serum levels by ELISA was performed as described previously [22]. Additional biological parameters assayed included *γ*-glutamyltranspeptidase (*γ*-GT; an indicator of liver function), alanine aminotransferase (ALT), aspartate aminotransferase (AST), total cholesterol, triglycerides, high-density lipoprotein (HDL), low-density lipoprotein (LDL), urea nitrogen, uric acid, fasting glucose, erythrocyte sedimentation rate at one hour, white blood cell count and type, platelet count, and hemoglobin. Prostate specific antigen (PSA) level was determined in subjects aged above 45 years old. The biochemical parameters were measured by using a Hitachi 917 analyzer and the hematological parameters at an autoanalyzer Pedra 120.

### 2.2. Statistical analysis

Results were expressed as mean (SD) or geometric mean (minimum-maximum). Differences between groups were examined by Student's *t*-test or Mann-Whitney *U*-test depending on the normality of the distribution. Pearson correlation analysis was used to determine possible correlations between variables. A log transformation was used for laboratory variables not fitting to a normal distribution. Wilcoxon signed rank test was employed to assess the difference on paired observations in intervention group. For comparisons, the two-tailed test was used with a type I error of *α* = 0.05. Data analyses were conducted by means of the SPSS for Windows 14.1.0 statistical package.

## 3. RESULTS

One out of three welders had a normal body mass index (BMI) (20–25 kg/m^2^), while 18.2% were obese (BMI >30 kg/m^2^). Only 19.1% of the subjects were nonsmokers and statistical analysis revealed a significant positive correlation between cigarette smoking and elevated triglyceride and hematocrit levels. Body mass index (BMI) besides age was also positively correlated to triglyceride level, uric acid, and *γ*-GT (Table 1). Welders had been welding for 11–25 days the previous month and for 180–240 days the previous year.

Cr blood levels in the first sampling ranged between 0.1–6.1 *μ*g/L (mean 0.91; geometric mean 0.64) and urine Cr levels ranged between 0.1–50.2 *μ*g/L (mean 1.33; geometric mean, 0.43) or 0.03–27.27 *μ*g/g creatinine (mean 0.87; geometric mean 0,25). In Table 1, relative data of subgroups are presented. ApoJ/CLU serum concentration ranged between 653 and 3075 (mean 1168; geometric mean 1129) (OD_492_). None of the hematological parameters, biochemical indicators, age, BMI, or smoking status showed any statistical significant relation with ApoJ/CLU levels. Urine Cr levels exhibited a weak association with ApoJ/CLU levels (*r* = 0.160, *P* = .157) raised when only SS welders were included in the analysis but it remained insignificant (*r* = 0.245, *P* = .097). Correlation of urine Cr corrected for creatinine and ApoJ/CLU levels was also not significant (*r* = 0.185, *P* = .101) but also raised in SS welders (*r* = 0.217, *P* = .143). Having observed that trend, we analyzed data from the first sampling concerning measurements of those welders known (from their detailed occupational history) to be involved recently (less than 10 weeks) in stainless steel welding. Indeed, when only this group (*n* = 17) was included in the analysis, the correlation coefficient (*r*) of ApoJ/CLU with urine Cr rose from 0.160 to 0.333 (*P* = .192) and with urine Cr corrected for creatinine rose from 0.245 to 0.453 (*P* = .068) but did not reach statistical significance (*P* = .206).

As we described previously, we implemented an intervention to lower Cr(VI) blood levels in order to study the regulation of ApoJ/CLU levels in human serum. All welders (*n* = 9) with blood Cr levels higher than 2 *μ*g/L were assigned to two intervention groups with different grade of intensity, followed a five-month intervention program which consisted of a significant differentiation (lowering) in the volume and amount of stainless steel-mediated welding. After this period, we recollected blood and urine and assayed the Cr and ApoJ/CLU serum levels. In both groups, we found a significant reduction in the Cr levels in all but one blood sample (Table 2). Urine levels exhibited various trends partly explained by the intensity of intervention, in addition to evidence that urine Cr reflects more recent exposure and it has greater variability [26].

**Figure 1:**
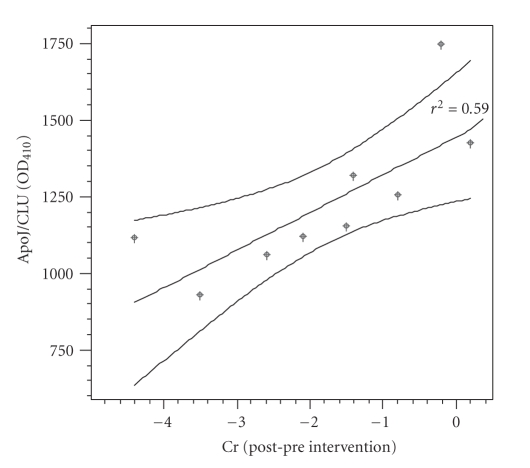
Relation of ApoJ/CLU levels and the reduction of chromium blood levels (micrograms/L) (lines represent 95% mean prediction interval).

Interestingly, we found that the reduction of Cr levels in blood due to this intervention was related to lower ApoJ/CLU serum levels (0.77, *P* = .009) (Figure 1). Higher intensity of intervention (lower exposure to hexavalent chromium) was also related to lower ApoJ levels in a statistical significant level (1042 versus 1362, *P* = .032).

The results of multivariate analysis modeling ApoJ/CLU levels have shown that blood Cr is the main determinant of ApoJ/CLU levels. Urine Cr holds a mild significant inverse association (negative *β*) reflecting perhaps differences in the kinetics between compartments (Table 3).

## 4. DISCUSSION

The unifying factor in determining toxicity and carcinogenicity for most, if not all, heavy metals including iron, copper, chromium, vanadium, cobalt, mercury, cadmium, and nickel is the generation of reactive oxygen and nitrogen species [27]. Metal-mediated formation of free radicals causes various modifications to DNA bases, enhanced lipid peroxidation and altered calcium and sulfhydryl homeostasis. Lipid peroxides, formed by the attack of radicals on polyunsaturated fatty acid residues of phospholipids, can further react with redox metals finally producing mutagenic and carcinogenic substances [27].

We have previously shown that welders and sandblasters, who are exposed to high levels of heavy metals, exhibited lower ApoJ/CLU serum levels as compared to other occupational groups [22]. This finding was unanticipated since exposure of normal human diploid fibroblasts to low noncytotoxic levels of Cr(VI) induced premature cellular senescence and resulted in the upregulation of the ApoJ/CLU protein [22]. ApoJ/CLU has a nearly ubiquitous expression pattern in human tissues and has been implicated in various physiological processes and in many severe physiological disturbance states including ageing, cancer progression, vascular damage, diabetes, kidney, and neuron degeneration [28]. Although unrelated in their etiology and clinical manifestation, these diseases represent states of increased oxidative stress [29, 30]. By combining these findings, we proposed recently that ApoJ/CLU upregulation during ageing or at age-related diseases does not correlate to chronological age, but it rather relates to increased oxidative damage which can be “sensed” by the regulatory elements of the CLU gene promoter [28].

Although chronic exposure may induce secondary molecular changes, which extend beyond the effects of oxidative stress (e.g., inflammation or initiation of tumor formation) [22], our current findings strengthen further the notion that the ApoJ/CLU is a sensitive biomarker of the organismal oxidative stress. More specifically, we report that the CLU serum levels correlate positively to the workers exposure to heavy metals and to Cr blood and urine concentration. Cr in blood has been shown to reflect occupational exposure to hexavalent chromium in stainless steel welding [31] which gives support to the argument that the significant reduction of Cr levels after the intervention is mainly due to the reduction of hexavalent chromium which is in turn assumed to be the responsible agent for the relation with ApoJ/CLU found in our study. However, it should be considered that regarding the exposure to other metals there might be some uncertainty in the assessment of the related biological effect. Lack of detailed knowledge of the kinetics of Cr in the blood and in the elimination compartments, especially urine, makes difficult to evaluate the correct time and site for sampling and the number of samples that should be taken. It is suggested that a longitudinal study in occupationally exposed participants besides Cr and ApoJ/CLU should also include the measurement of the levels of well-known markers of oxidative damage such as the products of lipid peroxidation [malondialdehyde(MDA)], DNA damage (modified bases such as 8-oxo-dG), and/or protein carbonylation in order to add firm basis to the proposed Cr-mediated oxidative stress in the cells of occupational groups with similar exposures as other studies have shown with various markers [32–37].

In any case, this preliminary study gives evidence that the human ApoJ/CLU gene is responsive to the acute oxidative stress induced by heavy metals as hexavalent chromium, and this finding may prove valuable during the monitoring and re-evaluation of the long-term workers health effects in certain occupational environments.

**Abbreviations**

Apolipoprotein J/Clusterin: ApoJ/CLU, (hexavalent) chromium: Cr(VI).

## Figures and Tables

**Table 1:** Individual characteristics and laboratory results in shipyard workers.

	SS welders	Sandblasters	Welders
	*n* = 47	*n* = 5	*n* = 28
	Mean sd	Mean sd	Mean sd
Age (years)	40.4 (9.0)	46.8 (6.6)	38.6 (10.8)
Duration of employment (years)	17.4 (11.5)	26.3 (7.8)	18.8 (12.6)
BMI (kg/m^2^)	29.1 (3.7)	28.0 (1.7)	26.5 (2.6)
Glucose (mg/dL)	89.2 (12.4)	98.4 (11.0)	85.0 (12.1)
Urea (mg/dL)	36.0 (8.8)	40.6 (10.3)	31.1 (6.7)
Creatinine	1.0 (0.1)	0.9 (0.1)	0.9 (0.1)
Uric acid (mg/dL)	5.5 (1.3)	5.0 (0.2)	5.4 (0.9)

Liver function indicators			

ALT (GPT) (U/L)	36.3 (18.4)	29.6 (12.1)	29.3 (13.2)
AST (GOT) (U/L)	22.7 (7.7)	18.4 (3.1)	21.0 (6.1)
GGT (U/L)	31.4 (15.5)	24.6 (6.4)	25.7 (17.4)

Lipid metabolism			

Cholesterol (mg/dL)	216.7 (43.8)	200 (35.3)	207.2 (35.8)
LDL (mg/dL)	138.4 (44.4)	131.2 (29.6)	134.7 (31.8)
HDL (mg/dL)	47.7 (8.5)	44.2 (4.6)	46.2 (7.9)
Triglycerides (mg/dL)	143.0 (122.7)	122.6 (53.7)	133.3 (54.1)

Hematology			

Hematocrit (%)	45.0 (2.6)	45.6 (3.3)	45.4 (2.7)
ESR (mm)	6.8 (5.6)	7.80 (7.1)	3.6 (2.0)
Leukocytes (WBC) (10^3^)	7.51 (2.08)	7.57 (0.55)	8.10 (1.99)
Neutocytes (10^3^)	4.23 (1.64)	4.29 (0.26)	4.62 (1.40)
Lymphocytes (10^3^)	2.63 (0.77)	2.67 (0.25)	2.77 (0.51)
Monocytes (10^3^)	0.43 (0.19)	0.40 (0.07)	0.44 (0.13)
Cr blood (*μ*g/L)	1.14 (1.16)	0.26 (0.13)	0.64 (0.68)
Cr urine (*μ*g/L)	1.84 (7.25)	0.20 (0.14)	0.68 (0.92)
Cr urine (*μ*g/g creatinine)	1.20 (4.08)	0.09 (0.04)	0.45 (0.98)
Clusterin/ApoJ (OD_492_)	1165.1 (338.1)	1140.9 (178.4)	1176.3 (274.9)

BMI = body mass index; ALT (GPT) = alanine aminotrasferase; AST (GOT) = aspartate aminotransferase; GGT = *γ*-glutamyltranspeptidase; LDL = low-density lipoprotein; HDL = high-density lipoprotein; ESR = erythrocyte sedimentation rate.

**Table 2:** Chromium blood and urine levels of welders entered worksite intervention (*n* = 9).

Welder	Age	Smoking status^1^	Cr blood (*μ*g/L)		Cr urine (*μ*g/g creatinine)		Cr urine (*μ*g/L)	
			Intervention		Intervention		Intervention	
			pre	post	pre	post	pre	post
1st	58	N	6.1	4.0	27.27	24.27	50.20	5.20
2nd	46	H	2.0	1.2	0.72	4.04	0.80	3.90
3rd	38	N	5.0	0.6	0.64	0.95	1.50	0.50
4th	40	M	2.9	0.3	1.03	0.43	1.10	0.20
5th	36	M	3.7	0.2	0.31	0.09	0.90	0.10
6th	37	L	2.5	2.7	0.65	2.40	1.20	1.60
7th	42	M	2.1	1.9	1.13	1.83	1.20	2.20
8th	35	L	3.0	1.6	1.94	2.64	3.10	3.20
9th	35	M	2.7	1.2	0.20	4.98	0.30	3.20

All mean	40.78		3.33	1.52^2^	3.77	4.62	6.70	2.23
(SD)	(7.40)		(1.38)	(1.22)	(8.83)	(7.54)	(16.33)	(1.79)

^1^N: nonsmokers; L: light (1–15 pack years); M: medium (16–30 pack years); H: heavy (>30 pack years)

^2^ *P* < .05, Wilcoxon signed ranks test.

**Table 3:** Linear regression results modeling for ApoJ/CLU serum levels.

Variable	Units of *β*	*β* coefficient (95% CI)	*P* value	Model *r* ^2^
MODEL 1				0.656

Cr blood	*μ*g/L	265.57 (71.38 to 1233.93)	.015	
Cr urine	*μ*g/g creatinine	− 32.53 (− 70.04 to − 7.02)	.024	

MODEL 2				0.590

pre/post intervention ΔCr blood	*μ*g/L	− 122.69 (− 213.69 to − 31.69)	.015	
